# Feelings Related to the COVID-19 Pandemic Among Patients Treated in the Oncology Clinics (Poland)

**DOI:** 10.3389/fpsyg.2021.647196

**Published:** 2021-04-27

**Authors:** Mateusz Grajek, Eliza Działach, Marta Buczkowska, Michał Górski, Elzbieta Nowara

**Affiliations:** ^1^Department of Public Health, Faculty of Health Sciences Bytom, Medical University of Silesia, Katowice, Poland; ^2^Department of Toxicology and Occupational Health Protection, Faculty of Health Sciences in Bytom, Medical University of Silesia, Katowice, Poland; ^3^Doctoral School, Medical University of Silesia, Katowice, Poland; ^4^Faculty of Health Sciences, Jan Dlugosz University, Czestochowa, Poland

**Keywords:** COVID-19, cancer, oncology, quality of life, fear

## Abstract

**Background:** The number of cancer patients is constantly growing. Both WHO and IARC report that this number may reach up to 24 million new diagnosed cases in the next two decades. The proposed treatment and especially the diagnosis can have a significant impact on an individual's approach to the disease, as well as on the patient's quality of life.

**Objectives:** The study aimed to assess the quality of life, feelings, and fear of cancer-treating oncological patients, before and during the COVID-19 pandemic.

**Material and Methods:** The study used the standardized WHOQOL quality of life questionnaire in a shortened version, the COVID-19 fear scale (FCV-19S), and the AIS disease acceptance scale (in terms of cancer-related sensations). The questionnaire survey was conducted among patients of cancer clinics (Poland). The study was conducted in two stages–before the COVID-19 pandemic (May 2019) and during the COVID-19 pandemic (May 2020). Data from 450 correctly completed questionnaires were analyzed statistically. The obtained data were statistically processed using the Kruskal-Wallis and Mann-Whitney U test (*p* = 0.05).

**Results:** Among the surveyed patients of the cancer clinic, the quality of life during the COVID-19 pandemic decreased by 2%, compared to the period before the pandemic. The frequency of negative feelings associated with cancer increased during the COVID-19 pandemic–by 11% more men, and 4.4% of women determined the frequency of negative feelings to be 2–3 times a week. The level of fear associated with COVID-19 was moderate (57.1%), with women having a higher level of fear (12.5% higher than men).

**Conclusion:** The development of the epidemic is very important in terms of public health. COVID-19 should be considered as one of the factors that bring about sudden changes in the mental health of the population, which may result from the dynamic development of this disease, dramatic media coverage, and own experiences. It has been shown that the sudden appearance of such a large stressor causes a decrease in patients' quality of life and an increase in negative feelings associated with chronic disease.

## Background

The number of cancer patients is constantly growing. Both WHO (World Health Organization) and IARC (International Agency for Research on Cancer) report that this number may reach up to 24 million new diagnosed cases over the next two decades (GLOBOCAN, 2012; Wild and Stewart, 2014). The proposed treatment, and especially the diagnosis, may significantly affect the individual's approach to the disease and also affect the patient's quality of life (Oleś, 2005). Quality of life conditioned health is a term that describes the physical, social, mental, psychological aspects of wellbeing and functioning evaluated by the patient or the impact of disease and treatment on functioning and life satisfaction (Schipper, 1990). The evaluation of patients' quality of life is one of the standards of medical care institutions, as negative life events, such as life-threatening cancer, affect the quality of human life more than positive events. According to reports conducted in Poland, 82.3% of patients rate their quality of life as below average, 96% of patients rate their general well-being very low and 93% rate their economic situation very low (Spieth and Harris, 1996).

The diagnosis of cancer is often a traumatic event. The physical and psychological consequences of the disease and anticancer treatment affect the existing functioning, may hinder or prevent many activities, cause reorganization of activities, abandonment of life's dreams and goals, revaluation of needs (Czapiński, 1992). However, the extent to which the determination of cancer and the process of treatment will change the functioning of an individual depends on various situational factors (e.g., social support) and subjective factors, such as the sense of competence, self-efficacy, mental resilience, the way of interpreting experiences, the strategy of dealing with the consequences of the disease (Sek, 1993). Good quality of life can be identified with subjective well-being, satisfaction, good adaptation to the situation, and the ability to perform social functions (Eiser and Morse, 2001). Studies have shown that a higher sense of the quality of life makes it easier to cope with the disease and affects the patient's mental comfort, while a higher level of health has a return effect on the subjective sense of the quality of life (Bańka, 2005; Gozdziewicz, 2005).

Oncological patients are a group with a high psychological burden (Glaser et al., 1987). More than 50% of cancer patients suffer from acute mental disorders, and about 33% meet the criteria of at least one mild mental disorder (Mehnert et al., 2014). The current pandemic may affect mental health due to the prevailing uncertainty, limitations, and social distance. Studies conducted so far have shown an increased mental burden associated with stress, anxiety, and depression since the beginning of sanitary restrictions (Rajkumar, 2020). Brooks et al. (2020) have studied the psychological effects of quarantine during a pandemic, pointing to the psychological burden of people unable to participate in public life. Considering the serious global threat and the impact the COVID-19 pandemic had on various aspects of life, Ahorsu et al. (2020) developed a scale to measure the fear of COVID-19 (FCV-19S). This scale has been used in many countries such as Bangladesh (Sakib et al., 2020), Iran (Alyami et al., 2020), Italy (Soraci et al., 2020), Turkey (Satici et al., 2020), Russia and Belarus (Reznik et al., 2020), Israel (Tzur Bitan et al., 2020), Paraguay (Barrios et al., 2020), Peru (Huarcaya-Victoria et al., 2020). In Polish literature, the usefulness of the scale was confirmed by the authors Pisula and Nowakowska (2020). At the beginning of March 2020, an epidemic was declared in Poland, which meant that all cultural, educational and medical facilities had to be closed and many planned treatments were canceled. This factor may have caused the level of fear in oncology patients to increase while their quality of life and acceptance of the disease deteriorated.

Because of the above, the study aimed to assess the quality of life, feelings, and fears of oncological patients treated for cancer, before and during the COVID-19 pandemic.

## Materials and Methods

The study used the standardized WHOQOL quality of life questionnaire in a shortened version^1^, the COVID-19 fear scale (FCV-19S) (Ahorsu et al., 2020; Pisula and Nowakowska, 2020), and the AIS disease acceptance scale^2^ (in terms of cancer-related sensations). On their basis, the respondents assessed the frequency of a given sensation (not at all, rarely, during the week, and every day). The questionnaire survey was conducted among patients of cancer clinics (Poland), with voluntary participation. All cancer patients participating in the study were cancer center patients who were diagnosed in 2019–2020 and did not require hospitalization for their condition. The survey was conducted in two stages–before the COVID-19 pandemic (May 2019) and during the COVID-19 pandemic (May 2020). The patients participating in the first and second phases of the study were the same individuals. Statistical analysis was performed on data from 450 correctly filled-in questionnaires. The first stage of the study was conducted stationary during patients' visits to the clinic, in the second stage a CAWI (Computer-Assisted Web Interview) web form was used. Conducting the study did not require the authors to obtain approval from a bioethics committee in light of the Physician and Dentist Profession Act of December 5, 1996, which provides a definition of medical experimentation.

The facilities selected for the study were selected by independent randomization. There are 15 oncology centers in Poland, five were selected by lot, one each from different regions of Poland (north, south, center, east, and west).

The WHOQOL quality of life questionnaire is self-description. It allows determining the degree of adaptation to the disease, the feeling of positive (satisfaction, joy) and negative (sadness, horror, the meaninglessness of life), as well as the quality of social contacts and support received. The respondent lists the factors that have the greatest influence on his or her quality of life (conducive and deteriorating quality of life). Based on the results of the WHOQOL questionnaire, the quality of life is assessed–from very bad to very good. Moreover, in collecting data on the frequency of negative feelings related to the disease, the following AIS scale characteristics were used: difficulty in adapting to the limitations imposed by the disease; inability to perform favorite activities; feeling of worthlessness and loneliness; feeling of dependence on the environment; feeling of being a burden for others. The respondents assessed on their basis how often a given feeling appears in their lives (never, occasionally, once a week, 2–3 times a week, daily).

Another tool used in the study was the FCV-19S scale according to Ahors (in Polish translation). Compliance with the statements (subscales) contained in the scale is scored from 1 to 5, according to the assumptions of the Likert scale, where one means disagreement with the statement and five means full agreement with the statement. The scale consists of seven sub-scales, from which a maximum of 35 points can be obtained. The final result is a percentage value (obtained after taking into account a coefficient of ~2,857), with the following verbal interpretation: 76–100% high level of fear; 56–75% moderate level of fear; 26–55% low level of related fear; <25% no COVID-19 related to fear. For the standardization test in the self-test, a Cronbach α 0.90 was obtained. The main purpose of the scale is to assess the respondent's emotions, which could indicate the level of fear or even anxiety associated with the possibility of SARS-CoV-2 infection. The reliability of the scale was confirmed in the pre-test and in other previously mentioned research projects (Alyami et al., 2020; Barrios et al., 2020; Huarcaya-Victoria et al., 2020; Pisula and Nowakowska, 2020; Reznik et al., 2020; Sakib et al., 2020; Satici et al., 2020; Soraci et al., 2020; Tzur Bitan et al., 2020).

The data obtained were developed in Statistica 13.0 by Statsoft, using the Kruskal-Wallis and Mann-Whitney U test (*p* = 0.05).

## Results

The research group consisted of 450 people with diagnosed cancer, who were patients of the oncological outpatient clinic at the time of the research. The average age of the respondents was 45 ± 6 years, 56% were women (*n* = 252) and 44% were men (*n* = 198). In terms of education, those with secondary education (34.2%) and vocational education (30.5%) were the largest group. Respondents with higher education were 23.8%), and those with primary education were 11.4%). In terms of education, those with secondary education (34.2%) and vocational education (30.5%) were the largest group. Respondents with higher education were 23.8%), and those with primary education were 11.4%). The most commonly reported cancer problem in this group was lung cancer (54.1%), cancer of the colon or other parts of the gastrointestinal tract (32.4%), other, including cancers of the head and neck region.

**Figure 1:**
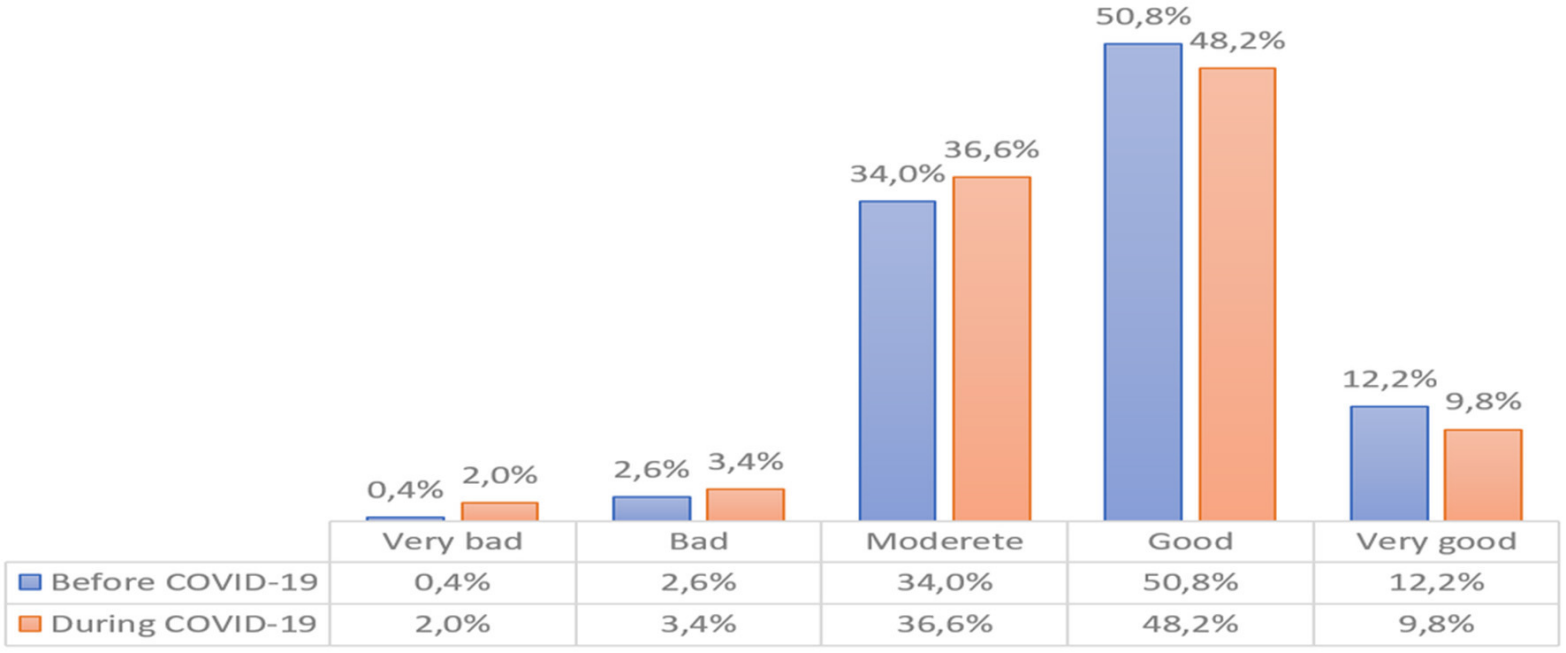
A summary evaluation of the quality of life in the study group (according to WHOQOL)—*N* = 450.

The first research problem concerned the level of quality of life of people treated with oncology. It is assumed that the quality of life includes the physical, mental, and behavioral spheres of human functioning. The examined persons made a subjective assessment of their quality of life. The results presented in Figure 1 show that the examined persons have a varied quality of life. In the first stage of the study (before the COVID-19 pandemic) about 12.2% of the subjects had a high quality of life and <0.4% low. In this period the largest groups were people with good (50.8%) and moderate quality of life (34.0%). During the COVID-19 pandemic, the quality of life changed. The number of people with very bad and bad quality of life increased−2.0 and 3.4%, respectively, while the number of people with good and very good quality of life decreased−48.2 and 9.8%, respectively. As in the period preceding the COVID-19 pandemic, the largest groups were people with good (48.2%) and moderate quality of life (36.6%). The average decrease in all ratings was about two percentage points between the analyzed periods (*p* < 0.05). The results are presented in Figure 1.

The WHOQOL questionnaire was also used in the self-assessment of the health condition of the examined patients. The obtained results indicate that women interpret their health condition worse than men (5.2% very bad and 28.8% bad vs. 2.2% very bad and 25.2 bad in the first stage of the study; 7.4% very bad and 33.2% bad vs. 5.0% very bad and 28.2% bad in the second stage). Very good and good health was characterized by an average of 3.5% and 25.0% of respondents at the pre-COVID-19 stage and 0.9 and 25.3% of respondents at the pandemic stage. It was observed that the results between the study periods differed–in the period before the pandemic the self-assessment of health condition was higher in the studied group of patients (*p* < 0.05)–Table 1.

**Table 1:** Self-assessment of health condition in the study group (according to WHOQOL)—*N* = 450.

**Value**	**Before COVID-19**		**During COVID-19**		***p*-value**
	**Female**	**Male**	**Female**	**Male**	
Very bad	5.2%	2.2%	7.4%	5.00%	<0.05
Bad	28.8%	25.2%	33.2%	28.20%	
Moderate	39.6%	36.0%	32.0%	41.80%	
Good	22.2%	27.8%	26.4%	24.20%	
Very good	4.2%	2.8%	1.0%	0.80%	

Selected subscales from the AIS questionnaire (see material and methods) were used to assess the frequency of bad sensations associated with cancer. Based on the results obtained it was observed that the frequency of negative feelings associated with cancer increased during the COVID-19 pandemic (*p* < 0.5) –Table 2.

**Table 2:** The frequency of negative feelings associated with the disease in the study group (according to AIS)—*N* = 450.

**Value**	**Before COVID-19**		**During COVID-19**		***P*-value**
	**Female**	**Male**	**Female**	**Male**	
Never	11.8%	20.6%	8.2%	7.0%	<0.05
Occasionally (2–3 times a month)	39.6%	44.6%	33.4%	32.2%	
One time a week	34.4%	26.6%	36.2%	38.4%	
2–3 times a week	11.8%	7.0%	16.2%	18.0%	
Daily	2.4%	1.2%	6.0%	4.4%	

Based on the results of the FCV-19S questionnaire it was found that the level of fear associated with COVID-19 was at a moderate level −20.0 points (57.1%). It was observed that the level of fear was higher among women (22.2 points −63.4%) than among men (17.8 points −50.8%)–*p* < 0.05. The highest point score was achieved by the discriminant “I am very afraid of SARS-CoV-2”−3.4 points, and the smallest “I can't sleep because I'm worried about getting a coronavirus” −2.4 points (Table 3).

**Table 3:** Level of fear of COVID-19 in the study group (according to FCV-19S)—*N* = 450.

**FCV-19S**	**X**	**SD**	**MIN**	**MAX**	**Me**	**Mo**
I am very afraid of SARS-CoV-2 (coronavirus)	3.4	0.8	2	5	3.5	3
I feel anxiety when I think of the coronavirus	3.2	0.6	2	5	3.5	3
My hands are sweating. when I think about the coronavirus	2.6	0.6	1	4	3	2
I'm afraid of losing my life because of the coronavirus	3.0	0.4	2	5	3.5	3
When I watch the news and learn about coronavirus-related stories on social media I get nervous or anxious	2.6	0.8	1	4	3	2
I can't sleep because I'm worried that I'm going to get a coronavirus	2.4	0.6	1	4	3	2
My heart is beating violently. when I think of a coronavirus infection	2.8	0.6	1	4	3	2
Total points	20.0					
Percentage	57.1%					
Word	Moderate fear level					

## Discussion

With the development of the COVID-19 pandemic worldwide, attention began to be paid to the special situation of chronically ill patients–including oncological patients. This was due both to a higher risk of coronavirus infection and its complications, for this group of patients, and the abandonment of diagnosis and treatment due to limited access to medical services. When the attention of decision-makers and the public was focused on new cases of coronavirus infections, the situation of many times larger group of oncological patients remained in the shadow.

According to the report “Oncology in COVID-19,”^3^ there is a huge decline in mammography and cytology during the pandemic. The number of patients diagnosed with cancer or suspected cancer has also been gradually decreasing compared to the previous year. Analyzing the absolute numbers, the decrease is even 30%. As a consequence, the number of specialist consultations also decreased–by about 25%. Concerning first-time benefits in oncological surgery in 2020 compared to 2019, the biggest drop was recorded in May, but the first reduction of benefits was already observed in March. Besides, the number of patients in clinical oncology hospitals has decreased by about a quarter and the implementation of benefits in the field of oncology drug programs has decreased by several percentage points. In the case of radiotherapy, including radical and palliative radiotherapy, the number of first consultations decreased by about 30% compared to 2019.

The described situations are reflected in indicators such as patients' quality of life, their feelings about the disease, and their fear of SARS-CoV-2 infection (COVID-19). In our study, we found that the quality of life during the COVID-19 pandemic decreased by 2% compared to the pre-pandemic period. The attitude to the disease also changed. Negative feelings related to the disease, such as problems with adapting to limitations, inability to perform favorite activities, feeling of worthlessness and loneliness, feeling addicted to the environment, feeling of being a burden to others; occurred much more often during the pandemic than in the period preceding it. Moreover, it was observed that the studied group is characterized by a moderate level of fear associated with COVID-19. These results are consistent with those of other researchers (Ahorsu et al., 2020; Alyami et al., 2020; Barrios et al., 2020; Huarcaya-Victoria et al., 2020; Pisula and Nowakowska, 2020; Reznik et al., 2020; Sakib et al., 2020; Satici et al., 2020; Soraci et al., 2020; Tzur Bitan et al., 2020). Previous studies (Holmes et al., 2020; Li J. et al., 2020) have also shown that the development of the epidemic is important in terms of public health. COVID-19 should be considered as one of the factors that bring about sudden changes in the mental health of the population, which may result from the dynamic development of this disease, dramatic media coverage, and own experiences. It has been shown that the sudden appearance of such a large stressor causes mood reduction, anxiety, and even psychotic disorders, which in extreme cases may lead to suicidal thoughts and suicidal actions (Li W. et al., 2020). It has also been shown that the lack of psychotherapeutic measures promotes the transformation of these symptoms into serious mental disorders, such as depression, anxiety disorders, or post-traumatic stress syndrome (Kim et al., 2015).

However, it is not only the current global epidemic situation that determines the deterioration of the quality of life in the studied group. The cancer process itself, which takes place in the patient's body, also has an impact. The studies conducted by Dendek et al. (2015) have shown that the most troublesome symptoms accompanying the disease are pain and sleep disorders. Moreover, based on the results of the Piskozub study, it was found that cancer affects the human personality, which manifests itself in low self-esteem, negative self-perception, the disappearance of the sense of life, anxiety, depression, and self-aggression (Piskozub, 2010). In Tobiasz-Adamczyk's study (Tobiasz-Adamczyk, 2012), the women examined after breast cancer treatment suffered from mood changes, feelings of anger, sadness, frustration, and after colorectal cancer treatment they additionally experienced discomfort following colostomy. In a study conducted by Juzwiszyn et al. (2016), most cancer patients are considered disabled (88%), of which 75% feel comfortable despite their disability. About 29% of the respondents felt the reluctance to take therapeutic measures. The motivating factors for undertaking treatment were the willingness to cure (73%) and fear for the family (35%).

Sigorski et al. (2020) conducted a study with a similar theme. The authors determined the level of fear of the SARS-CoV-2 virus among patients of five Polish oncology centres. A total of 306 patients participated in this study. The mean FCV-19S score in this group was 18.5 ± 7.44, which is lower than that of the oncology patients included in their own study (20.1 ± 4.7). A similar mean FCV-19S score of 18.48 ± 5.32 was obtained in their study by Parlapani et al. (2020); however, their work focused on elderly people living in Greece.

In a study by Fujita et al. (2020) involving 165 patients treated at the National Hospital Organization Kyoto Medical Center, 9.1% of lung cancer patients experienced anxiety and chose to delay treatment during the COVID-19 pandemic. In contrast, the work of Tzur Bitan et al. (2020), which analyzed the records of 3,661 patients undergoing chemotherapy, proves that during the COVID-19 pandemic the treatment delay rate increased significantly (from 11.6 to 14.2%). Fear of the pandemic was among the reasons for postponing chemotherapy, with 13.6%, while this percentage dropped to 4.6% after the introduction of telemedicine.

The COVID-19 pandemic resulted in the closure of many aid centers, foundations, or associations, where patients could exchange views and observations on the therapy. Patients were often alone with their illness, cut off from the family, due to a total ban on visits, which reduced their mood and also affected the development of depressive disorders. All this may adversely affect the success of oncological therapy.

## Conclusions

1. Among the examined patients of the cancer clinic, the quality of life during the COVID-19 pandemic decreased by 2%, compared to the period before the pandemic.
2. The frequency of negative feelings associated with cancer increased during the COVID-19 pandemic−11% more men and 4.4% more women defined the frequency of negative feelings as 2–3 times a week.
3. The level of fear associated with COVID-19 was moderate (57.1%), with women having a higher level of fear (12.5% higher than men).

## Data Availability Statement

The raw data supporting the conclusions of this article will be made available by the authors, without undue reservation.

## Author Contributions

MGr: idea and methods. ED and EN: material. MB: redaction. MGó: methods. All authors contributed to the article and approved the submitted version.

## Conflict of Interest

The authors declare that the research was conducted in the absence of any commercial or financial relationships that could be construed as a potential conflict of interest.

## Footnotes

^1^WHOQOL–BREF: https://www.who.int/substance_abuse/research_tools/en/polish_whoqol.pdf [on-line: 28.12.2020].

^2^AIS: http://pcdit.wsei.lublin.pl/wp-content/uploads/2014/11/AIS-Skala-Akceptacji-Choroby.pdf [on-line: 28.12.2020].

^3^Onkologia w czasie COVID-19: http://onkologia2025.pl/userfiles/321321321/onkologia/COVID19/Onkologia%20w%20czasach%20COVID-19%20raport.pdf [on-line: 28.12.2020].
